# Early Biomarkers of Altered Renal Function and Orthostatic Intolerance During 10-day Bedrest

**DOI:** 10.3389/fphys.2022.858867

**Published:** 2022-04-20

**Authors:** Grazia Tamma, Annarita Di Mise, Marianna Ranieri, Mariangela Centrone, Maria Venneri, Mariagrazia D’Agostino, Angela Ferrulli, Boštjan Šimunič, Marco Narici, Rado Pisot, Giovanna Valenti

**Affiliations:** ^1^ Department of Biosciences, Biotechnologies and Biopharmaceutics, University of Bari, Bari, Italy; ^2^ Institute of Kinesiology Research, Science and Research Centre, Koper, Slovenia; ^3^ Department of Biomedical Sciences, University of Padova, Padova, Italy

**Keywords:** bedrest, vasopressin, copeptin, aquaporin-2 (AQP2), osteopontin, adrenomedullin, calcium, kidney

## Abstract

Exposure to actual or simulated microgravity results in alterations of renal function, fluid redistribution, and bone loss, which is coupled to a rise of urinary calcium excretion. We provided evidence that high calcium delivery to the collecting duct reduces local Aquaporin 2 (AQP2)-mediated water reabsorption under vasopressin action, thus limiting the maximal urinary concentration to reduce calcium saturation. To investigate early renal adaptation into simulated microgravity, we investigated the effects of 10 days of strict bedrest in 10 healthy volunteers. We report here that 10 days of inactivity are associated with a transient, significant decrease (day 5) in vasopressin (copeptin) paralleled by a decrease in AQP2 excretion, consistent with an increased central volume to the heart, resulting in reduced water reabsorption. Moreover, bedrest caused a significant increase in calciuria secondary to bone demineralization paralleled by a decrease in PTH. Urinary osteopontin, a glycoprotein exerting a protective effect on stone formation, was significantly reduced during bedrest. Moreover, a significant increase in adrenomedullin (day 5), a peptide with vasodepressor properties, was observed at day 5, which may contribute to the known reduced orthostatic capacity post-bedrest. We conclude that renal function is altered in simulated microgravity and is associated with an early increase in the risk of stone formation and reduced orthostatic capacity post-bedrest within a few days of inactivity.

## Introduction

It is well established that during space flight, weightlessness causes muscle loss, a significant increase of serum calcium, mostly released by bones, a decrease in intestinal calcium reabsorption, and relevant hypercalciuria (Iwamoto et al., 2005; Smith et al., 2015). Moreover, fluid redistribution and abnormal renal functions also occur. Hypercalciuria is a determinant factor associated with an increased risk for the development of kidney stones, nephrocalcinosis, and osteoporosis (Figueres et al., 2020). Importantly, hypercalciuric patients display a reduced urinary concentrating ability because the physiological response to the action of the antidiuretic hormone vasopressin is depressed. This impairment is associated with a significant reduction of the AQP2 excretion, known to be proportional to its expression in the collecting duct lumen (Pisitkun et al., 2004; Tamma et al., 2014). Low body negative pressure (LBNP) and bedrest are established models of simulating gravitational changes. In a previous study of 35 days of continuous bedrest, we found a progressive decrease in AQP2 excretion that reached the lowest value at day 14 (Tamma et al., 2014). Interestingly, the transient reduction of urinary AQP2 was paralleled by a transient increase in the calciuria revealing an inverse correlation between AQP2 excretion and urinary calcium level (Tamma et al., 2014). At the moment, however, early biomarkers associated with microgravity or simulated microgravity remain still unknown and need to be identified and characterized.

Calcium homeostasis is tightly modulated by several hormones. Parathyroid hormone (PTH) plays a pivotal role in controlling serum calcium concentration. Specifically, the calcium-sensing receptor (CaSR) which is highly expressed in the parathyroid glands, can sense tiny alterations in serum Ca^2+^ levels. Physiologically, low serum Ca^2+^ concentration stimulates PTH release that results in calcium uptake from the intestine, kidney, and bone (Riccardi and Valenti, 2016). At the renal level, PTH stimulates the production of the active form of Vitamin D which exerts numerous activities in the bone and promotes calcium reabsorption from the intestine. At the systemic level, the procalcemic actions of PTH are antagonized by specific antagonists including calcitonin. At a local level, osteopontin (OPN) counteracts the actions of PTH. Osteopontin is a multifunctional phospho-glycoprotein expressed in osteoblasts where it blocks the synthesis of the hydroxyapatite crystal and bone mineralization (Kitahara et al., 2003; Ono et al., 2008). In a model of simulated microgravity, horizontal rotation of osteoblasts for 24, 48, and 72 h results in a significant drop in the expression of OPN (Wan et al., 2005). Interestingly, in the kidney, reduction of OPN decreases the risk of renal stone formation (Hamamoto et al., 2010; Tsuji et al., 2014). The hydration state condition can reduce kidney crystal formation by modulating the intrarenal OPN expression (Lee et al., 2016).

Beyond the effects on muscles, bones, kidneys, the endocrine and immune systems, exposure to microgravity or simulated microgravity (SMG) modifies vascular contractility often resulting in hypotension. Rats subjected to hindlimb unweighting, another model of simulated microgravity, showed a high level of adrenomedullin (ADM) (Neri et al., 2002). ADM is a 52-amino acid peptide playing a key role in controlling blood pressure in the short term. In this respect, we have recently found an increase in ADM in response to central hypovolemia in male volunteers subjected to LBNP (Goswami et al., 2019). Here, a study of 10 days of strict bedrest was undertaken to identify early biomarkers associated with altered renal physiology. In particular, copeptin was detected and used as a stable marker of the hormone vasopressin which is involved in controlling the fluid balance. Moreover, urinary AQP2, calciuria, OPN, cystatin C as a marker of GFR and KIM1 as a marker of oxidative stress were evaluated as well.

## Materials and Methods

### Experimental Protocol

This study was supported by the Italian Space Agency (ASI) project “MARS-PRE Bed Rest SBI 2019”, approved by the National Ethical Committee of the Slovenian Ministry of Health (Ref. number: 0120-304/2019/9) and performed following the standard set by the Declaration of Helsinki. All participants were informed about the aims, procedures, and potential risks of the investigations before written consents were obtained.

Ten young healthy, recreationally active males (age, 23 ± 5 years; height, 1.81 ± 0.04 m; body mass (BM), 77.5 ± 10.0 kg; body mass index (BMI), 23.5 ± 2.5 kg m^–2^) were enrolled in this study. Female were excluded from the study to avoid confounding physiological conditions related to the menstrual cycle. Participants’ body mass and body mass index estimated in pre and post bed rest conditions in a parallel study revealed a slight but significant decrease (Zuccarelli et al., 2021).

Subjects underwent a medical screening before the study. Exclusion criteria were: regular smoking, habitual use of drugs, blood clotting defects, history of deep vein thrombosis, neuromuscular, metabolic, and cardiovascular disease conditions, previous history of embolism, inflammatory diseases, psychiatric disorders, epilepsy, and presence of ferromagnetic implants.

Each subject was evaluated before and after 10 days of strict horizontal bedrest and during bedrest no deviations from the lying position, muscle stretching, or static contraction were allowed. Participants were constantly controlled using continuous closed-circuit television surveillance with supervision by researchers and medical staff. Subjects arrived at the hospital three days before bedrest for pre-bedrest measurements. Post-bedrest measurements were carried out immediately after day 10 of bedrest for two days. During the two days post-bedrest subjects stayed in a wheelchair or in bed between the measurements performed. Subjects consumed an individually controlled, standardized diet and were allowed to drink water *ad libitum*. Diet was generally controlled on the level of macronutrients (60% carbohydrates, 25% fats and 15% proteins). Blood draw was taken every day before the breakfast at 7 in the morning. Blood pressure was monitored continuously by Task Force Monitor (TFM, CNSystems, Graz, Austria). Orthostatic blood pressure was measured during supine to stand test immediately after waking up after 10 days of bedrest.

### Urinary AQP2 Measurements by ELISA (Enzyme-Linked Immunosorbent Assay)

For each subject, spot urine samples were taken every day in the morning fasting, between 7.00 and 7.15. Collected urine samples were immediately frozen and stored at −20°C. Urinary excretion of AQP2 was measured in the urine specimens by ELISA as previously described (Goswami et al., 2019). It is known that AQP2 is excreted in the urine through the endocytosis-multiple vesicular body (MVB)-exosome pathway (Pisitkun et al., 2004) representing a useful biomarker for the renal response to vasopressin (Valenti et al., 2000). Approximately 3% of AQP2 in the kidney is excreted daily and is proportional to the AQP2 reaching the apical plasma membrane in response to vasopressin (Rai et al., 1997). Urine samples were added with protease inhibitors (1 mM PMSF, 2 mg/ml leupeptin, 2 mg/ml pepstatin A) and cellular debris were removed spinning at 3,000 rpm for 10 min at 4°C. 5 µL of urine sample were diluted to 50 µL in PBS containing 0.01% SDS, placed in a MaxiSorp 96-well microplate and incubated overnight at 4°C. In parallel wells, decreasing concentrations (1,000, 500, 400, 300, 200, 100, and 50 pg/50 µL) of a synthetic peptide reproducing the last 15 amino acids of the C-terminal region of human AQP2 were incubated as internal standard. Each sample was analyzed in triplicate. Wells were then washed with washing buffer (PBS-0.1% Tween20) and incubated with blocking solution (PBS−3% BSA) at 37°C for 1 h. 10 µg of affinity-purified anti-AQP2 antibodies were diluted in blocking solution (final antibody dilution 1:1,500) and 50 µL of the solution was added to each well and incubated for 2 h at 37°C. Wells were then washed four times with washing solution and incubated with secondary goat anti-rabbit antibodies conjugated to horseradish peroxidase for 1 h at 37°C. After five washes, 50 µL of the substrate solution [2,29-azino-bis(3-ethylbenzthiazoline-6-sulfonic acid)] was added to each well and incubated for 30 min in the dark at room temperature. Absorbance was measured with a microplate reader (model iMark, Bio-Rad Laboratories, Milan, Italy) at 415 nm. Urinary AQP2 excretion was expressed as fmol/mg urine creatinine.

### Biomarkers Measurement

Biomarkers were measured from serum. Blood samples were collected into chilled plastic tubes with disodium-EDTA and aprotinin. The tubes were placed on ice before centrifugation at 1,600 × g for 15 min at 4°C to collect the serum and stored at − 80°C immediately until further processing. Copeptin (CPP) levels were quantified by an ELISA kit (Cusabiotechnology LLC) which provides a sensitivity lower than 19.5 pg/ml. KIM-1, Osteopontin (OPN) were measured by ELISA kit (R&D Systems), following the manufacturer’s instructions. The minimum detectable dose for KIM-1, and OPN ranged from 0.003–0.046 ng/ml, 0.030–0.227 ng/ml and 0.006–0.024 ng/ml, respectively. Adrenomedullin (ADM) quantification was performed using an ELISA kit (MyBioSource) with a sensitivity as low as 10.4 pg/ml. All the ELISA assays performed are commercially available.

### Urinary Calcium, Sodium, Potassium and Serum Parathyroid Hormone Determination

Urinary Calcium, Sodium and Potassium, and Serum PTH, calcium, sodium, potassium, phosphorus and 25-OH Vitamin D were measured by Service Laboratory using an automated system.

### Statistical Analysis

All values are reported as means ± S.E.M. Statistical analysis was performed using Ordinary one-way ANOVA followed by Dunnett’s multiple comparisons test with *p* < 0.05 considered statistically different. For Serum Copeptin and Adrenomedullin was used One sample *t*-test with *p* < 0.05 considered statistically different.

## Results

### Effect of Bedrest on Fluid Regulating Hormones, Aquaporin-2, and electrolytes

The major aim of the study was the identification of early biomarkers of altered renal function and orthostatic intolerance in simulated microgravity on the ground. To this end, 10 healthy males (age, 23 ± 5 years) participated in the experiment in the Hospital of Ankaran (Slovenia). The biomarkers for renal function were tested either in the urine or in the blood collected daily during the bedrest. Immobilization or exposure to microgravity induces several alterations of renal function including fluid redistribution (Drummer et al., 2002; Gaspare De Santo et al., 2005). It is known that vasopressin is the key hormone regulating water homeostasis. Vasopressin was measured as copeptin, a stable surrogate marker of vasopressin (Shrabani, 2015).

**FIGURE 1:**
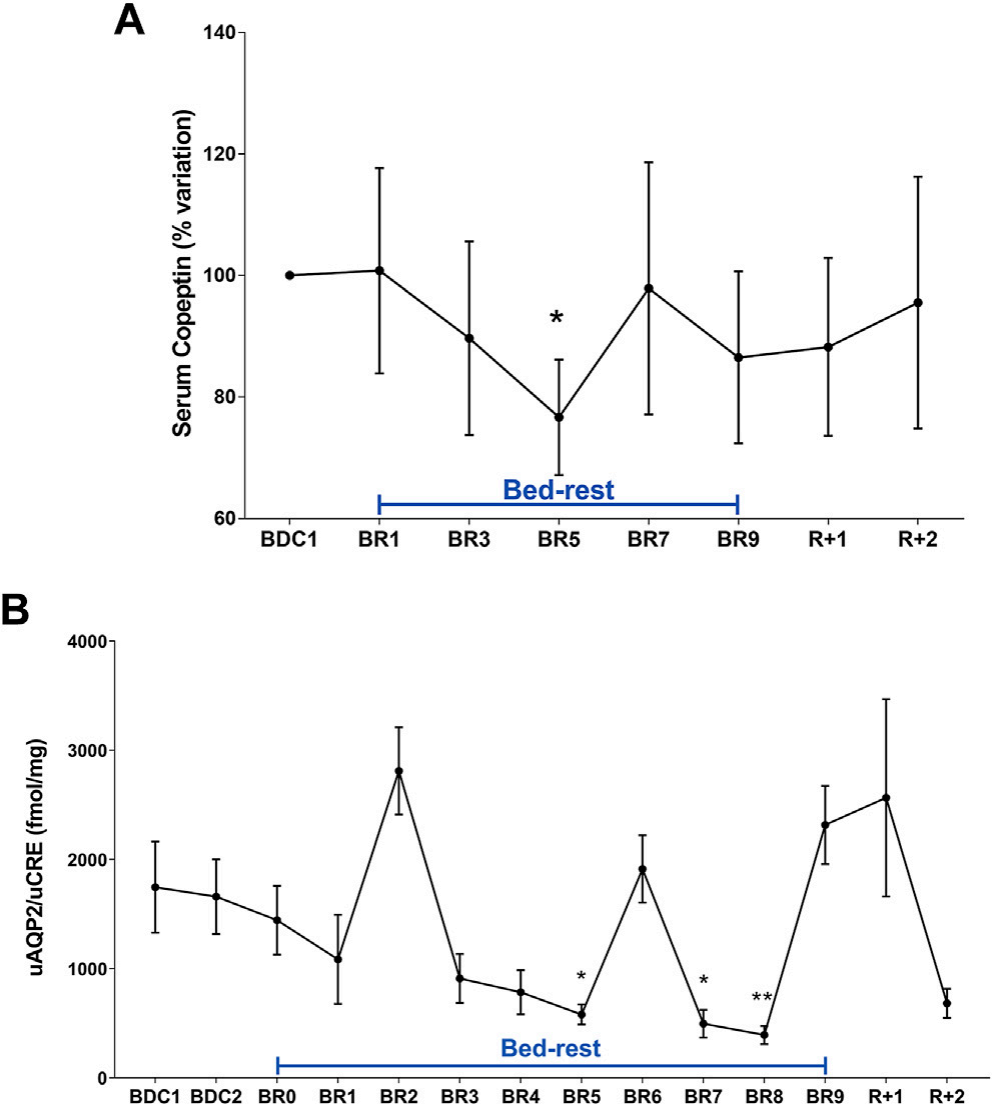
**(A)** Serum Copeptin (% variation versus BDC1) during 10-day bedrest. Data are expressed as mean ± S.E.M. Statistical analysis was done using One Sample *t* test (*p < 0.05 for BR5 vs. BDC1); **(B)** urinary AQP2 excretion normalized to Creatinuria (fmol/mg) during 10-day bedrest. Data are expressed as mean ± S.E.M. Statistical analysis was done using Ordinary one-way Anova test (*p < 0.05 for BR5 and BR7 vs. BDC1; **p < 0.01 for BR8 vs. BDC1).

Copeptin was found significantly decreased at day 5 (Figure 1A). This data correlated with the significant reduction in urinary AQP2, considered a biomarker for collecting duct responsiveness to vasopressin, also observed at day 5 (Figure 1B). Except day 6, AQP2 excretion was significantly reduced also at day 7 and 8 in agreement with data obtained in a previous 35‐day bedrest study performed in the same facility, showing a significant decrease in AQP2 excretion (measured in 24 h urine samples) starting from around day 6–7 until day 14 (Tamma et al., 2014). The reason why we don’t see a parallel significant decrease in copeptin after day 5 despite a significant reduction in AQP2 excretion may be due to the fact that, physiologically, vasopressin action in the collecting duct is counteracted by Calcium Sensing Receptor signaling activated by high urinary calcium concentrations (Riccardi and Valenti, 2016; Ranieri et al., 2018; Ranieri, 2019). Therefore, despite vasopressin (copeptin) levels returned normal after day 5, urinary AQP2 levels and in turn the rate of water reabsorption can remain reduced, reflecting attenuation of vasopressin effect.

**FIGURE 2:**
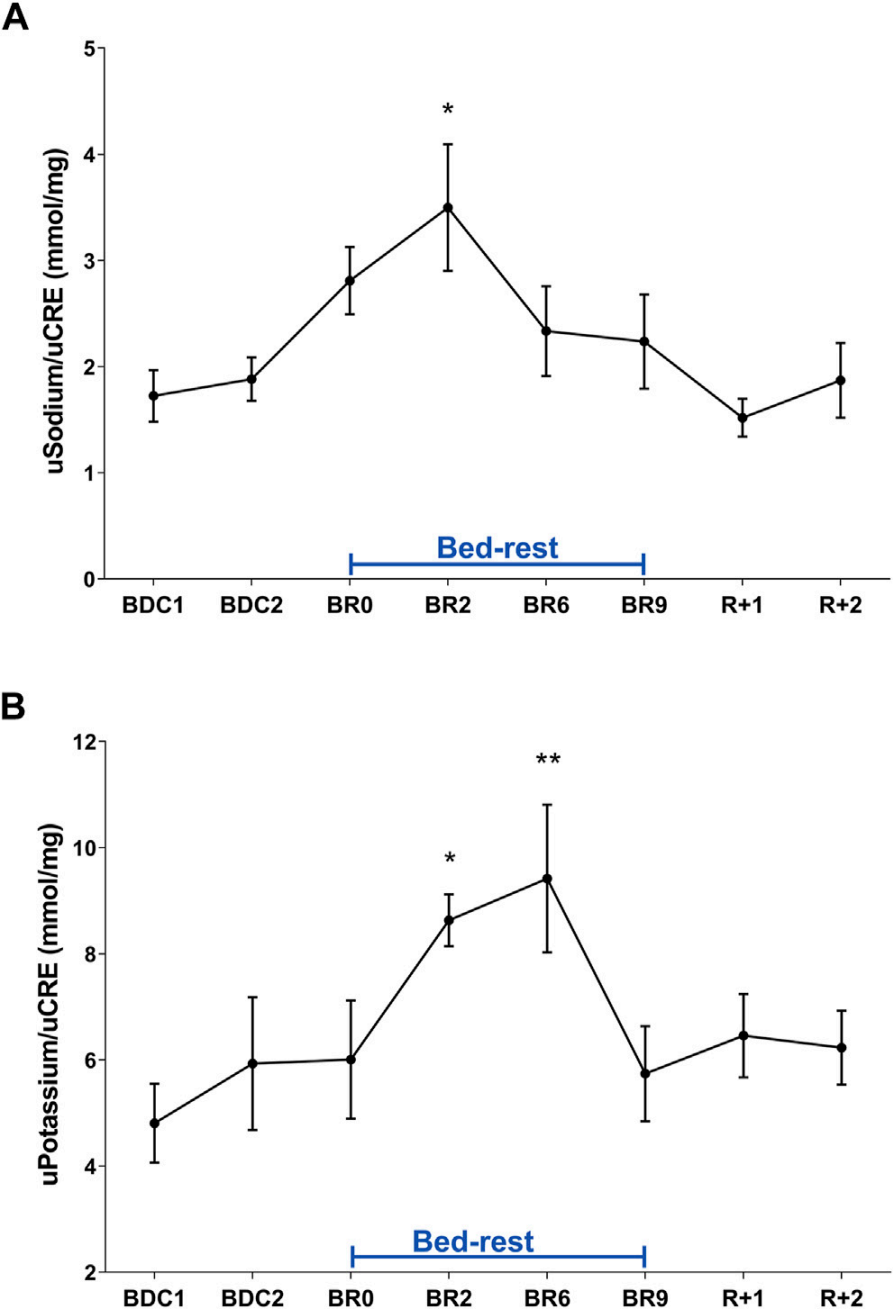
**(A)** Urinary Sodium excretion normalized to Creatinuria (mmol/mg) during 10-day bedrest. Data are expressed as mean ± S.E.M. Statistical analysis was done using Ordinary one-way Anova test (*p < 0.05 for BR2 vs. BDC1); **(B)** urinary Potassium excretion normalized to Creatinuria (mmol/mg) during 10-day bedrest. Data are expressed as mean ± S.E.M. Statistical analysis was done using Ordinary one-way Anova test (*p < 0.05 for BR2 vs. BDC1; **p < 0.01 for BR6 vs. BDC1).

These data suggest that adaptation to a supine posture causes an increase in venous return and in central volume inducing higher renal water excretion to reduce volume, a process mediated by a reduction in vasopressin release and then in AQP2-mediated water reabsorption. Besides vasopressin, another hormone, the atrial natriuretic peptide (ANP) can also respond to increased central volume as previously shown in a bedrest study (Moro et al., 2007) and in a head-out water immersion study (Valenti et al., 2006). In addition to water, sodium regulation in humans is sensitive to posture change in gravitational stress (Norsk, 1992). Urinary excretion of sodium and potassium during bedrest was measured daily (Figures 2A,B). Urinary sodium excretion significantly increased early during bedrest reaching the maximal value at day 2 followed by a progressive decline and return to pre-bedrest levels after the recovery (Figure 2A). Potassium excretion also showed a transient significant increase reaching the maximal value at day 6 before returning to normal pre-bedrest values during the recovery phase (Figure 2B).

### Effect of Bedrest on Urinary Calcium and PTH

**FIGURE 3:**
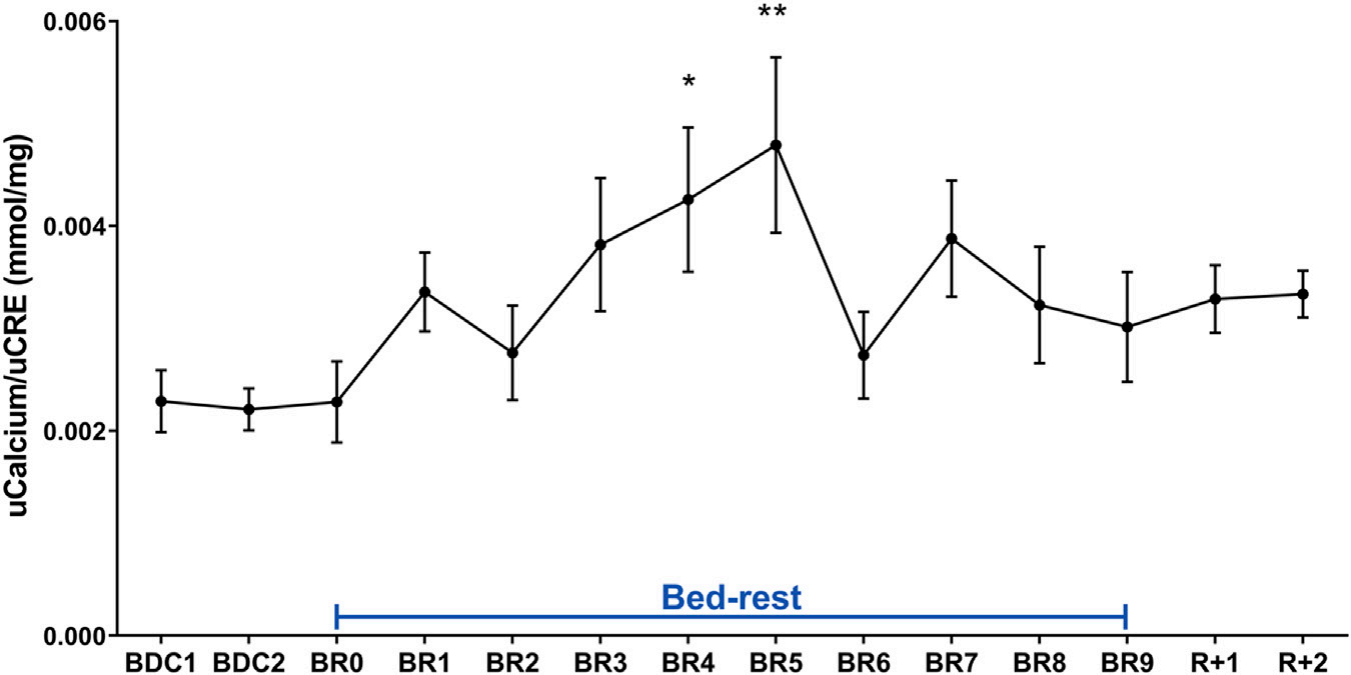
Urinary Calcium excretion normalized to Creatinuria (mmol/mg) during 10-day bedrest. Data are expressed as mean ± S.E.M. Statistical analysis was done using Ordinary one-way Anova test (*p < 0.05 for BR4 vs. BDC1; **p < 0.01 for BR5 vs. BDC1).

**FIGURE 4:**
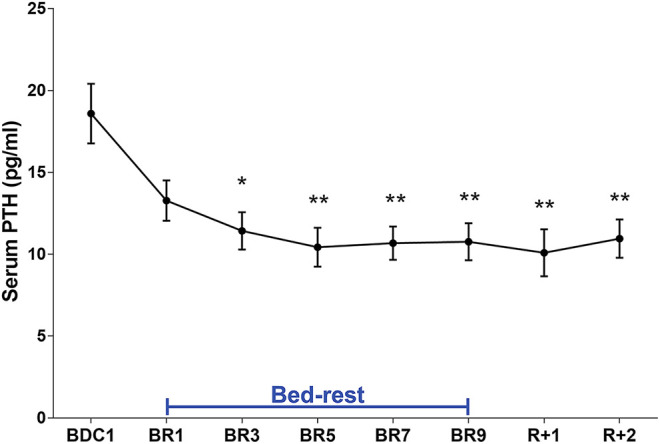
Serum PTH (pg/ml) during 10-day bedrest. Data are expressed as mean ± S.E.M. Statistical analysis was done using Ordinary one-way Anova test (*p < 0.05 for BR3 vs. BDC1; **p < 0.01; for BR5, BR7, BR9, R+1 and R+2 vs. BDC1).

Analysis of urinary calcium excretion revealed an early increase in calciuria during immobilization consistent with some previous bedrest studies (Baecker et al., 2003; Watanabe et al., 2004; Armbrecht et al., 2010; Bilancio et al., 2013; Tamma et al., 2014). As previously described, the increase in calciuria during bedrest is paralleled by an increase in calcemia which peaked at day 7, then decreased to below-baseline values (Bilancio et al., 2013). Calciuria had a clear tendency to raise since day 1, reaching a statistically significant value on days 4- and 5 (Figure 3). In parallel with the observed hypercalciuria, parathyroid hormone (PTH) progressively and significantly declined during bedrest reaching a significantly lower value already at day 3 compared to pre-bedrest and did not recover in the two post bedrest days (Figure 4). The sustained downregulation of the PTH during immobilization supports the view that bone resorption accounts for increases in urinary calcium excretion.

### Plasma Levels of Calcium, Sodium, Potassium, Phosphorus and 25-OH Vitamin D During Bedrest

Table 1 shows plasma levels of calcium, sodium, potassium, phosphorus and 25-OH Vitamin D during bedrest before (PRE), during (BR) and after (POST) bed rest. Calcemia significantly increased during bedrest and then decreased to levels not significantly different from pre-bedrest, in line with the described time course of calciuria. Electrolytes potassium, sodium and phosphorus also significantly increased during bedrest and then decreased to levels not significantly different from pre-bedrest. In contrast, 25-OH Vitamin D levels progressively and strongly decreased during bedrest and remained significantly low after bed rest. The changes in plasma phosphorus and Vitamin D axis during immobilization can be secondary to the observed sustained downregulation of the PTH.

**TABLE 1:** Serum Electrolytes and 25OH-Vit D of participants before (PRE), during (BR) and after (POST) 10-day horizontal bed rest.

	PRE	BR	POST	*p* value
Ca^2+^ (mg/dl)	9.37 ± 0.216	10.54 ± 0.197***	9.94 ± 0.120	0.0003
K^+^(mmol/L)	4.33 ± 0.096	4.84 ± 0.126**	4.68 ± 0.104	0.006
Na^+^(mmol/L)	137.0 ± 1.314	151.6 ± 1.707***	150.9 ± 2.965^###^	<0.0001
P (mg/dl)	3.23 ± 0.077	3.76 ± 0.107**	3.72 ± 0.104^##^	*0.001; ^#^0.003
25OH-Vit D (ng/ml)	66.14 ± 1.901	34.16 ± 2.173***	33.53 ± 2.296^###^	<0.0001

Values are means ± S.E.M.; *p* values different from PRE

### Urinary Kidney Injury Molecule-1 (KIM-1) as Potential Biomarkers of Renal Disfunction

We next explored additional urinary biomarkers known to be associated with adverse kidney outcomes in other settings. KIM-1 is considered a urine biomarker of kidney tubular health (Greenberg et al., 2021). Urinary KIM-1 did not change significantly during bedrest (data not shown).

### Effect of Bedrest on Urinary Osteopontin

**FIGURE 5:**
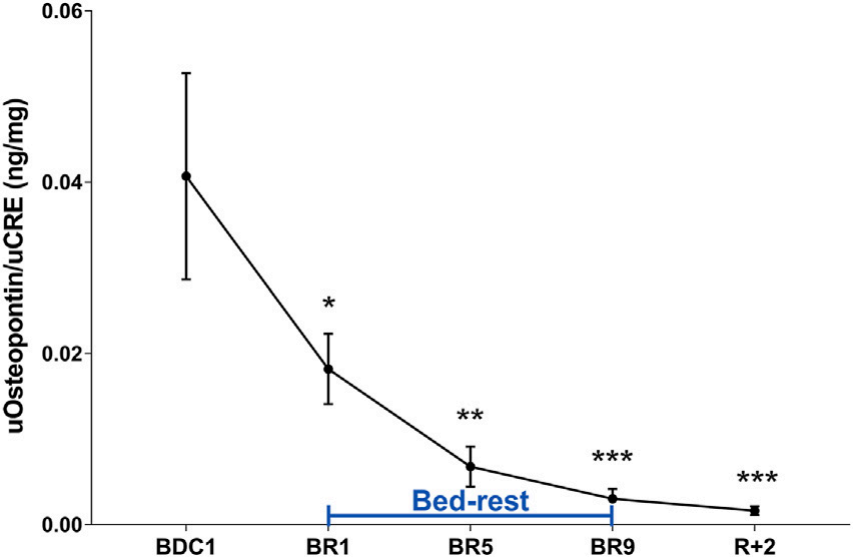
Urinary Osteopontin (OPN) excretion normalized to Creatinuria (ng/mg) during 10-day bedrest. Data are expressed as mean ± S.E.M. Statistical analysis was done using Ordinary one-way Anova test (*p < 0.05 for BR1 vs. BDC1; **p < 0.01 for BR5 vs. BDC1; ***p < 0.001 for BR9 and R+2 vs. BDC1).

OPN is considered to be an inhibitor of biomineralization (Franzen and Heinegard, 1985; Fisher et al., 2001). Interestingly OPN is present in kidneys and secreted in the urine (Kaleta, 2019). In the kidney, OPN has protective in renal stone formation by inhibiting aggregation of calcium oxalate crystals (Shiraga et al., 1992). Analysis of urinary OPN revealed a strong early reduction in urinary OPN, highly significant at day 5 until the end of the bedrest (Figure 5). OPN remained reduced in the 2 days after recovery (Figure 5).

### Bedrest and Orthostatic Intolerance

**FIGURE 6:**
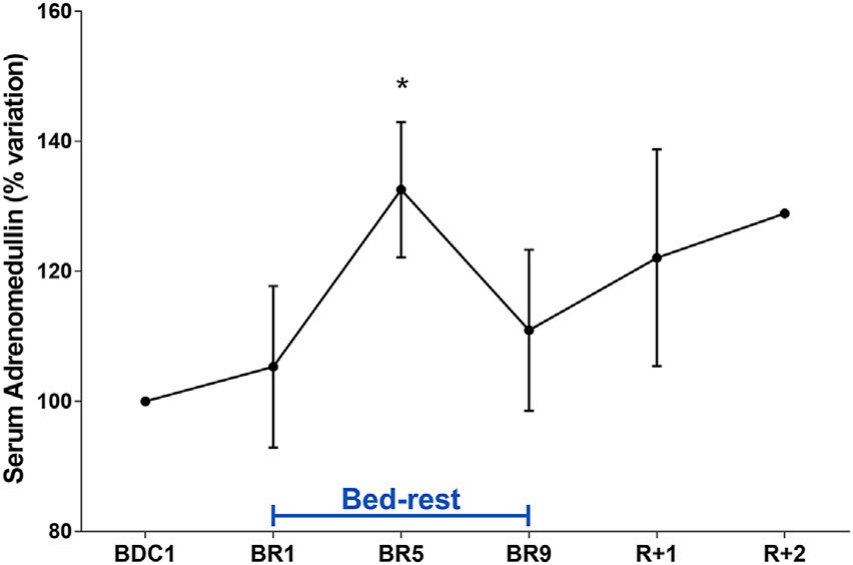
Serum Adrenomedullin (ADM) (% variation vs. BDC1) during 10-day bedrest. Data are expressed as mean ± S.E.M. Statistical analysis was done using One Sample *t* test (*p < 0.05 for BR5 vs. BDC1).

Orthostatic intolerance is a common consequence of bedrest (Barbic et al., 2019). Moreover, post-flight hypovolemia has been reported in conjunction with post-flight orthostatic intolerance (Blomqvist et al., 1994). We have previously reported that plasma concentrations of adrenomedullin (ADM), a peptide with vasodepressor properties, rise significantly during orthostatic challenge after 21-day bedrest (O’shea et al., 2015). We show here that, compared to pre-bedrest condition, ADM was statistically significantly higher at day 5, with no apparent return to pre-bedrest value during recovery (Figure 6). This is the first report showing an early increase in ADM during bedrest indicating that bedrest appears to affect ADM levels which may contribute to the reduced orthostatic capacity post-bedrest within a few days of immobilization.

Of interest, orthostatic blood pressure measured during supine to stand test immediately after 10 days of bedrest showed that 30% of participants fainted during supine-to-stand test, and the average time to faint was 63 ± 11 s. Others maintained orthostatic tolerance for 5 min. Interestingly, ADM values evaluated in subjects who to faint during supine-to-stand test were about 50% higher at day 5 with respect to the other subjects (167.5 ± 15.8% at peak vs. 117.6 ± 5.95%). Moreover, ADM values in these subjects remained elevated during the recovery time. These data support the indication that ADM can be a promising early biomarker of orthostatic intolerance.

## Discussion

The purpose of this study was to identify early biomarkers of altered renal function and orthostatic intolerance during 10-day bedrest, an approach that may prove useful for the identification of early biomarkers of renal abnormality and for the development of appropriate countermeasures.

Bedrest represents a valuable experimental model to mimic the effects of microgravity on Earth (Mukai and Ohshima, 2012; Goswami and Valenti, 2020). However, few studies investigated the human functional decline during 10-day bedrest and most of these focused on skeletal muscle, peripheral nerve adaptations, aerobic capacity, and bone alterations (Center et al., 2007; Mukai and Ohshima, 2012; Manganotti et al., 2021).

Some alterations of renal function including fluid redistribution, bone loss induced hypercalciuria, renal stone risk, reduced plasma volume, secondary to immobilization or bedrest have been described (Drummer et al., 2002; Gaspare De Santo et al., 2005; Cavalier et al., 2013; Smith et al., 2015). More specifically, in a previous study, we evaluated the effects of 35-day bedrest on changes in urinary calcium, modulation of the vasopressin-regulated water channel aquaporin-2, and blood hematocrit the latter used as an index of changes in plasma volume (Tamma et al., 2014). Thirty-five days of bedrest represents a prolonged period of inactivity that may actually mimic the chronic adaptations occurring in microgravity. Under these conditions we observed bone demineralization and a transient increase in urinary calcium followed by a transient decrease in urinary AQP2 excretion, which can reduce the ability to concentrate urine, causing plasma volume reduction (Tamma et al., 2014). The present study, however, shows that alterations in kidney handling of water and electrolytes occur already in the first 7–10 days of bedrest, underlining the importance of a careful early evaluation of physiological renal adaptation to simulated microgravity.

In this study, we investigated the effect of 10 days of strict bedrest in 10 healthy volunteers. The main results obtained can be summarized as follows: 10 days of bedrest are associated with: *a.* transient and a significant decrease (day 5) in vasopressin (copeptin) paralleled by a decrease in AQP2 excretion; *b.* significant increase in calciuria secondary to bone demineralization paralleled by a decrease in PTH; *c.* significantly reduction during bedrest in urinary osteopontin, a glycoprotein exerting a protective effect on stone formation; *d.* significant increase in adrenomedullin (day 5), a peptide with vasodepressor properties, which may contribute to the known reduced orthostatic capacity post-bedrest.

The adoption of a supine body position in bedrest can be considered a typical hypervolemia model in human physiology. It is known that this condition induces diuretic and natriuretic renal response that can be ascribed to the shift of blood volume from the legs to the upper part of the body (Drummer et al., 2000; Winkelman, 2009). Consistent with an early renal counter-response to hypervolemia, our data show a significant reduction in vasopressin (copeptin) at day 5 paralleled by a reduction of urinary AQP2 excretion, considered a biomarker of the renal response to vasopressin, as well as to significant early increase in sodium excretion. All these alterations are expected to result in a prompt physiological reaction to hypervolemic insult promoting water and sodium excretion to reduce circulating volume. AQP2 excretion during 10 days appeared however rather variable, possibly due to differences among the subjects participating to the study. However our data are in agreement with data obtained in our previous 35-day bedrest study showing a significant decrease in AQP2 excretion starting from around day 7 until day 14 (Tamma et al., 2014).

Bedrest also increases calcium excretion, and the source of calcium is bone (Winslow, 1985; Cavalier et al., 2013; Tamma et al., 2014). Due to increased calcium excretion, prolonged bedrest is associated with a risk of renal stone formation, primarily, stones of calcium oxalate and calcium phosphate (Okada et al., 2008). In this study, we provide a novel observation that bedrest causes a significant increase in calciuria secondary to bone demineralization paralleled by a decrease in PTH levels, which are significant already at days 3–5 of bedrest along with a decrease in 25-OH Vitamin D. Although we did not measure bone resorption markers, a previous work (Bilancio et al., 2013) confirmed that within the first 10 days of bedrest a stable increase in urinary deoxypyridoline, a marker of bone resorption was observed supporting the view that bone resorption accounts for increase in urinary calcium and a sustained PTH suppression.

Of note, this is accompanied by a significant decrease in urinary osteopontin starting from day 1 and remaining significantly lower compared to pre-bedrest in the two days of recovery.

Osteopontin is a glycoprotein known to be involved in biomineralization and remodeling (Franzen and Heinegard, 1985; Fisher et al., 2001). Osteopontin is also found in kidneys (in the thick ascending limbs of the loop of Henle and distal nephrons) and is secreted in the urine (Kaleta, 2019). Several studies provided evidence for a protective role of osteopontin in renal stone formation. Indeed, it has been shown that osteopontin can inhibit the nucleation and aggregation of calcium oxalate crystals *in vitro* (Shiraga et al., 1992; Worcester and Beshensky, 1995; Asplin et al., 1998). In line with these observations, patients with kidney stones have lower urinary excretion of osteopontin than healthy controls (Hoyer et al., 1995; Huang et al., 2003).

Interestingly, during the 10-day bedrest we observed a strong early reduction in urinary osteopontin, significant at day 5 and remained reduced up to the end of the bedrest and also during recovery. It has been reported that osteopontin is under control of PTH (Kaleta, 2019) which is also reduced during the 10-day bedrest. These data suggest that bedrest is associated with an early increased risk of stone formation. To our knowledge, this is the first report investigating osteopontin as a possible early biomarker of risk of renal stone formation under immobilization.

Another crucial aspect investigated in this study is orthostatic intolerance. In a previous study, we evaluated the role of adrenomedullin, a peptide with vasodepressor properties, in reduced orthostatic tolerance following 21-day bedrest immobilization (O’shea et al., 2015). Plasma adrenomedullin is known to rise during the orthostatic challenge. Specifically, measurements of baseline (supine), presyncope, and recovery levels of adrenomedullin in 8 healthy men, before and after 21-day of −6° head-down bedrest (HDBR), (O’shea et al., 2015) demonstrated that mean orthostatic tolerance decreased from 22.17 min before HDBR to 13.81 min following HDBR. In addition to the vasodilating effects that may contribute to orthostatic intolerance, adrenomedullin has actions on fluid and electrolyte homeostasis causing natriuresis and diuresis, (Ebara et al., 1994; Taylor and Samson, 2002). Accordingly, the kidney is thought to be a major producer of adrenomedullin and causes natriuresis and diuresis (Jougasaki and Burnett, 2000). Moreover, in isolated perfused tubules it has been reported that adrenomedullin inhibits osmotic water permeability associated with a decreased trafficking of AQP2 to the plasma membrane (Ma et al., 2020).

Of interest, we show here that, compared to pre-bedrest condition, adrenomedullin had an early clear tendency to increase since the first day of bedrest, reaching a peak on day 5, with no apparent return to pre-bedrest value during recovery.

Moreover, the observed increase in adrenomedullin, having vasodilator properties, is very well paralleled by the significant decrease in vasopressin having vasoconstrictor properties also at day 5, two neuropeptides both acting on vascular system causing a peripheral hypotensive effect. This is the first report showing an early increase in adrenomedullin during bedrest, indicating that bedrest appears to immediately affect adrenomedullin levels which may contribute to the reduced orthostatic capacity post-bedrest within a few days of immobilization.

Taken together our results indicate that 10 days of bedrest affect body fluid regulation consistent with an increased central volume to the heart associated with an early increase in the risk of stone formation that may be monitored by calcium excretion and osteopontin reduction. Moreover, we suggest a possible additional role of adrenomedullin in reducing water reabsorption contributing to the hypotensive and reduced orthostatic capacity associated with immobilization.

This study demonstrates the utility of novel plasma and urinary biomarkers of altered renal function and orthostatic intolerance after 10-day bedrest. These results will help prompt interventions to prevent or ameliorate the altered renal function that occurs in patients subjected to forced immobilization even for a few days.

## Data Availability

The original contributions presented in the study are included in the article/Supplementary Material, further inquiries can be directed to the corresponding authors.

## Abbreviations

AQP2, Aquaporin 2; PTH, Parathyroid hormone; OPN, Osteopontin; ADM, Adrenomedullin; KIM-1, Urinary kidney injury molecule-1

## References

[B1] Armbrecht G. Belavý D. L. Gast U. Bongrazio M. Touby F. Beller G. (2010). Resistive Vibration Exercise Attenuates Bone and Muscle Atrophy in 56 Days of Bed Rest: Biochemical Markers of Bone Metabolism. Osteoporos. Int. 21, 597–607. 10.1007/s00198-009-0985-z PubMed Abstract | 10.1007/s00198-009-0985-z | Google Scholar 19536451

[B2] Asplin J. R. Arsenault D. Parks J. H. Coe F. L. Hoyer J. R. (1998). Contribution of Human Uropontin to Inhibition of Calcium Oxalate Crystallization. Kidney Int. 53, 194–199. 10.1046/j.1523-1755.1998.00739.x PubMed Abstract | 10.1046/j.1523-1755.1998.00739.x | Google Scholar 9453018

[B3] Baecker N. Tomic A. Mika C. Gotzmann A. Platen P. Gerzer R. (2003). Bone Resorption Is Induced on the Second Day of Bed Rest: Results of a Controlled Crossover Trial. J. Appl. Physiol. 95, 977–982. 10.1152/japplphysiol.00264.2003 PubMed Abstract | 10.1152/japplphysiol.00264.2003 | Google Scholar 12909597

[B4] Barbic F. Heusser K. Minonzio M. Shiffer D. Cairo B. Tank J. (2019). Effects of Prolonged Head-Down Bed Rest on Cardiac and Vascular Baroreceptor Modulation and Orthostatic Tolerance in Healthy Individuals. Front. Physiol. 10, 1061. 10.3389/fphys.2019.01061 PubMed Abstract | 10.3389/fphys.2019.01061 | Google Scholar 31507438 PMC6716544

[B5] Bilancio G. Lombardi C. Pisot R. Mekjavic I. B. De Santo N. G. Luciano M. G. (2013). Effects of Prolonged Immobilization on Sequential Changes in mineral and Bone Disease Parameters. Am. J. Kidney Dis. 61, 845–847. 10.1053/j.ajkd.2012.10.015 PubMed Abstract | 10.1053/j.ajkd.2012.10.015 | Google Scholar 23177703

[B6] Blomqvist C. G. Buckey J. C. Gaffney F. A. Lane L. D. Levine B. D. Watenpaugh D. E. (1994). Mechanisms of post-flight Orthostatic Intolerance. J. Gravit. Physiol. 1, P122–P124. PubMed Abstract | Google Scholar 11538739

[B7] Cavalier E. Delanaye P. Moranne O. (2013). Variability of New Bone mineral Metabolism Markers in Patients Treated with Maintenance Hemodialysis: Implications for Clinical Decision Making. Am. J. Kidney Dis. 61, 847–848. 10.1053/j.ajkd.2012.12.013 PubMed Abstract | 10.1053/j.ajkd.2012.12.013 | Google Scholar 23357107

[B8] Center J. R. Bliuc D. Nguyen T. V. Eisman J. A. (2007). Risk of Subsequent Fracture after Low-Trauma Fracture in Men and Women. JAMA 297, 387–394. 10.1001/jama.297.4.387 PubMed Abstract | 10.1001/jama.297.4.387 | Google Scholar 17244835

[B9] Drummer C. Gerzer R. Baisch F. Heer M. (2000). Body Fluid Regulation in Μ-Gravity Differs from that on Earth: an Overview. Pflügers Arch. - Eur. J. Physiol. 441, R66–R72. 10.1007/s004240000335 10.1007/s004240000335 | Google Scholar 11200983

[B10] Drummer C. Valenti G. Cirillo M. Perna A. Bellini L. Nenov V. (2002). Vasopressin, Hypercalciuria and Aquaporin - the Key Elements for Impaired Renal Water Handling in Astronauts? Nephron 92, 503–514. 10.1159/000064111 PubMed Abstract | 10.1159/000064111 | Google Scholar 12372931

[B11] Ebara T. Miura K. Okumura M. Matsuura T. Kim S. Yukimura T. (1994). Effect of Adrnomedullin on Renal Hemodynamics and Functions in Dogs. Eur. J. Pharmacol. 263, 69–73. 10.1016/0014-2999(94)90524-x PubMed Abstract | 10.1016/0014-2999(94)90524-x | Google Scholar 7821363

[B12] Figueres L. Hourmant M. Lemoine S. (2020). Understanding and Managing Hypercalciuria in Adults with Nephrolithiasis: Keys for Nephrologists. Nephrol. Dial. Transpl. 35, 573–575. 10.1093/ndt/gfz099 PubMed Abstract | 10.1093/ndt/gfz099 | Google Scholar 31219589

[B13] Fisher L. W. Torchia D. A. Fohr B. Young M. F. Fedarko N. S. (2001). Flexible Structures of SIBLING Proteins, Bone Sialoprotein, and Osteopontin. Biochem. Biophysical Res. Commun. 280, 460–465. 10.1006/bbrc.2000.4146 PubMed Abstract | 10.1006/bbrc.2000.4146 | Google Scholar 11162539

[B14] Franzén A. Heinegård D. (1985). Isolation and Characterization of Two Sialoproteins Present Only in Bone Calcified Matrix. Biochem. J. 232, 715–724. 10.1042/bj2320715 PubMed Abstract | 10.1042/bj2320715 | Google Scholar 4091817 PMC1152943

[B15] Gaspare De Santo N. Cirillo M. Valenti G. Perna A. Anastasio P. Drummer C. (2005). Renal Function in Space: the Link between Osteoporosis, Hypercalciuria, and Aquaporins. J. Ren. Nutr. 15, 183–188. 10.1053/j.jrn.2004.09.011 PubMed Abstract | 10.1053/j.jrn.2004.09.011 | Google Scholar 15648031

[B16] Goswami N. Reichmuth J. Di Mise A. Brix B. Roessler A. Centrone M. (2019). Comparison between Men and Women of Volume Regulating Hormones and Aquaporin-2 Excretion Following Graded central Hypovolemia. Eur. J. Appl. Physiol. 119, 633–643. 10.1007/s00421-018-4053-2 PubMed Abstract | 10.1007/s00421-018-4053-2 | Google Scholar 30564880

[B17] Goswami N. Valenti G. (2020). Human Systems Physiology, 6. Google Scholar

[B18] Greenberg J. H. Abraham A. G. Xu Y. Schelling J. R. Feldman H. I. Sabbisetti V. S. (2021). Urine Biomarkers of Kidney Tubule Health, Injury, and Inflammation Are Associated with Progression of CKD in Children. Jasn 32, 2664–2677. 10.1681/asn.2021010094 PubMed Abstract | 10.1681/asn.2021010094 | Google Scholar 34544821 PMC8722795

[B19] Hamamoto S. Nomura S. Yasui T. Okada A. Hirose M. Shimizu H. (2010). Effects of Impaired Functional Domains of Osteopontin on Renal crystal Formation: Analyses of OPN Transgenic and OPN Knockout Mice. J. Bone Miner Res. 25, 2712–2723. 10.1359/jbmr.090520 PubMed Abstract | 10.1359/jbmr.090520 | Google Scholar 19453257

[B20] Hoyer J. R. Otvos L. Jr. Urge L. (1995). Osteopontin in Urinary Stone Formation. Ann. NY Acad. Sci. 760, 257–265. 10.1111/j.1749-6632.1995.tb44636.x PubMed Abstract | 10.1111/j.1749-6632.1995.tb44636.x | Google Scholar 7785900

[B21] Huang H.-S. Ma M.-C. Chen C.-F. Chen J. (2003). Lipid Peroxidation and its Correlations with Urinary Levels of Oxalate, Citric Acid, and Osteopontin in Patients with Renal Calcium Oxalate Stones. Urology 62, 1123–1128. 10.1016/s0090-4295(03)00764-7 PubMed Abstract | 10.1016/s0090-4295(03)00764-7 | Google Scholar 14665375

[B22] Iwamoto J. Takeda T. Sato Y. (2005). Interventions to Prevent Bone Loss in Astronauts during Space Flight. Keio J. Med. 54, 55–59. 10.2302/kjm.54.55 PubMed Abstract | 10.2302/kjm.54.55 | Google Scholar 16077253

[B23] Jougasaki M. Burnett J. C. Jr. (2000). Adrenomedullin as a Renal Regulator Peptide. Nephrol. Dial. Transpl. 15, 293–295. 10.1093/ndt/15.3.293 PubMed Abstract | 10.1093/ndt/15.3.293 | Google Scholar 10692508

[B24] Kaleta B. (2019). The Role of Osteopontin in Kidney Diseases. Inflamm. Res. 68, 93–102. 10.1007/s00011-018-1200-5 PubMed Abstract | 10.1007/s00011-018-1200-5 | Google Scholar 30456594

[B25] Kitahara K. Ishijima M. Rittling S. R. Tsuji K. Kurosawa H. Nifuji A. (2003). Osteopontin Deficiency Induces Parathyroid Hormone Enhancement of Cortical Bone Formation. Endocrinology 144, 2132–2140. 10.1210/en.2002-220996 PubMed Abstract | 10.1210/en.2002-220996 | Google Scholar 12697722

[B26] Lee S.-Y. Lee S.-J. Piao H.-L. Yang S.-Y. Weiner I. D. Kim J. (2016). Hydration Status Affects Osteopontin Expression in the Rat Kidney. J. Vet. Sci. 17, 269–277. 10.4142/jvs.2016.17.3.269 PubMed Abstract | 10.4142/jvs.2016.17.3.269 | Google Scholar 26645343 PMC5037293

[B27] Ma F. Chen G. Rodriguez E. L. Klein J. D. Sands J. M. Wang Y. (2020). Adrenomedullin Inhibits Osmotic Water Permeability in Rat Inner Medullary Collecting Ducts. *Cells* 9. 10.3390/cells9122533 10.3390/cells9122533 | Google Scholar PMC7760190 33255239

[B28] Manganotti P. Buoite Stella A. Ajcevic M. Di Girolamo F. G. Biolo G. Franchi M. V. (2021). Peripheral Nerve Adaptations to 10 Days of Horizontal Bed Rest in Healthy Young Adult Males. Am. J. Physiology-Regulatory, Integr. Comp. Physiol. 321, R495–R503. 10.1152/ajpregu.00146.2021 10.1152/ajpregu.00146.2021 | Google Scholar 34318712

[B29] Moro C. Pillard F. De Glisezinski I. Crampes F. Thalamas C. Harant I. (2007). Atrial Natriuretic Peptide Contribution to Lipid Mobilization and Utilization during Head-Down Bed Rest in Humans. Am. J. Physiology-Regulatory, Integr. Comp. Physiol. 293, R612–R617. 10.1152/ajpregu.00162.2007 PubMed Abstract | 10.1152/ajpregu.00162.2007 | Google Scholar 17553844

[B30] Mukai C. Ohshima H. (2012). Space Flight/bedrest Immobilization and Bone. In-Flight Exercise Device to Support a Health of Astronauts. Clin. Calcium 22, 1887–1893. CliCa121218871893. PubMed Abstract | CrossRef Full Text | Google Scholar 23187082

[B31] Neri G. Bova S. Malendowicz L. K. Mazzocchi G. Nussdorfer G. G. (2002). Simulated Microgravity Impairs Aldosterone Secretion in Rats: Possible Involvement of Adrenomedullin. Am. J. Physiology-Regulatory, Integr. Comp. Physiol. 283, R832–R836. 10.1152/ajpregu.00099.2002 PubMed Abstract | 10.1152/ajpregu.00099.2002 | Google Scholar 12228051

[B32] Norsk P. (1992). Gravitational Stress and Volume Regulation. Clin. Physiol. 12, 505–526. 10.1111/j.1475-097x.1992.tb00355.x PubMed Abstract | 10.1111/j.1475-097x.1992.tb00355.x | Google Scholar 1395444

[B33] O'shea D. Lackner H. K. Rössler A. Green D. A. Gauger P. Mulder E. (2015). Influence of Bed Rest on Plasma Galanin and Adrenomedullin at Presyncope. Eur. J. Clin. Invest. 45, 679–685. 10.1111/eci.12455 PubMed Abstract | 10.1111/eci.12455 | Google Scholar 25912957

[B34] Okada A. Ohshima H. Itoh Y. Yasui T. Tozawa K. Kohri K. (2008). Risk of Renal Stone Formation Induced by Long-Term Bed Rest Could Be Decreased by Premedication with Bisphosphonate and Increased by Resistive Exercise. Int. J. Urol. 15, 630–635. 10.1111/j.1442-2042.2008.02067.x PubMed Abstract | 10.1111/j.1442-2042.2008.02067.x | Google Scholar 18479353

[B35] Ono N. Nakashima K. Rittling S. R. Schipani E. Hayata T. Soma K. (2008). Osteopontin Negatively Regulates Parathyroid Hormone Receptor Signaling in Osteoblasts. J. Biol. Chem. 283, 19400–19409. 10.1074/jbc.m800005200 PubMed Abstract | 10.1074/jbc.m800005200 | Google Scholar 18417476 PMC3762377

[B36] Pisitkun T. Shen R.-F. Knepper M. A. (2004). Identification and Proteomic Profiling of Exosomes in Human Urine. Proc. Natl. Acad. Sci. 101, 13368–13373. 10.1073/pnas.0403453101 PubMed Abstract | 10.1073/pnas.0403453101 | Google Scholar 15326289 PMC516573

[B37] Rai T. Sekine K. Kanno K. Hata K. Miura M. Mizushima A. (1997). Urinary Excretion of Aquaporin-2 Water Channel Protein in Human and Rat. Jasn 8, 1357–1362. 10.1681/asn.v891357 PubMed Abstract | 10.1681/asn.v891357 | Google Scholar 9294826

[B38] Ranieri M. (2019). Renal Ca2+ and Water Handling in Response to Calcium Sensing Receptor Signaling: Physiopathological Aspects and Role of CaSR-Regulated microRNAs. Int. J. Mol. Sci. 20. 10.3390/ijms20215341 PubMed Abstract | 10.3390/ijms20215341 | Google Scholar PMC6862519 31717830

[B39] Ranieri M. Zahedi K. Tamma G. Centrone M. Di Mise A. Soleimani M. (2018). CaSR Signaling Down‐regulates AQP2 Expression via a Novel microRNA Pathway in Pendrin and NaCl Cotransporter Knockout Mice. FASEB j. 32, 2148–2159. 10.1096/fj.201700412rr PubMed Abstract | 10.1096/fj.201700412rr | Google Scholar 29212817

[B40] Riccardi D. Valenti G. (2016). Localization and Function of the Renal Calcium-Sensing Receptor. Nat. Rev. Nephrol. 12, 414–425. 10.1038/nrneph.2016.59 PubMed Abstract | 10.1038/nrneph.2016.59 | Google Scholar 27157444

[B41] Shiraga H. Min W. Vandusen W. J. Clayman M. D. Miner D. Terrell C. H. (1992). Inhibition of Calcium Oxalate crystal Growth *In Vitro* by Uropontin: Another Member of the Aspartic Acid-Rich Protein Superfamily. Proc. Natl. Acad. Sci. 89, 426–430. 10.1073/pnas.89.1.426 PubMed Abstract | 10.1073/pnas.89.1.426 | Google Scholar 1729712 PMC48250

[B42] Shrabani M. A. G. (2015). A Novel Stress Neurohormone Copeptin: Its Potential Role in Diagnosis and Prognosis of Various Diseases. World J. Pharm. Res. 4, 1927–1948. Google Scholar

[B43] Smith S. M. Heer M. Shackelford L. C. Sibonga J. D. Spatz J. Pietrzyk R. A. (2015). Bone Metabolism and Renal Stone Risk during International Space Station Missions. Bone 81, 712–720. 10.1016/j.bone.2015.10.002 PubMed Abstract | 10.1016/j.bone.2015.10.002 | Google Scholar 26456109

[B44] Tamma G. Di Mise A. Ranieri M. Svelto M. Pisot R. Bilancio G. (2014). A Decrease in Aquaporin 2 Excretion Is Associated with Bed Rest Induced High Calciuria. J. Transl Med. 12, 133. 10.1186/1479-5876-12-133 PubMed Abstract | 10.1186/1479-5876-12-133 | Google Scholar 24885203 PMC4035801

[B45] Taylor M. M. Samson W. K. (2002). Adrenomedullin and the Integrative Physiology of Fluid and Electrolyte Balance. Microsc. Res. Tech. 57, 105–109. 10.1002/jemt.10055 PubMed Abstract | 10.1002/jemt.10055 | Google Scholar 11921361

[B46] Tsuji H. Shimizu N. Nozawa M. Umekawa T. Yoshimura K. De Velasco M. A. (2014). Osteopontin Knockdown in the Kidneys of Hyperoxaluric Rats Leads to Reduction in Renal Calcium Oxalate crystal Deposition. Urolithiasis 42, 195–202. 10.1007/s00240-014-0649-0 PubMed Abstract | 10.1007/s00240-014-0649-0 | Google Scholar 24619192 PMC4979578

[B47] Valenti G. Fraszl W. Addabbo F. Tamma G. Procino G. Satta E. (2006). Water Immersion Is Associated with an Increase in Aquaporin-2 Excretion in Healthy Volunteers. Biochim. Biophys. Acta (Bba) - Biomembranes 1758, 1111–1116. 10.1016/j.bbamem.2006.03.029 10.1016/j.bbamem.2006.03.029 | Google Scholar 16764820

[B48] Valenti G. Laera A. Pace G. Aceto G. Lospalluti M. L. Penza R. (2000). Urinary Aquaporin 2 and Calciuria Correlate with the Severity of Enuresis in Children. Jasn 11, 1873–1881. 10.1681/asn.v11101873 PubMed Abstract | 10.1681/asn.v11101873 | Google Scholar 11004218

[B49] Wan Y. M. Ma Y. J. Zhang X. Y. Zeng B. Wang H. H. Li Y. H. (2005). Effects of Rotation on Osteonectin and Osteopontin mRNA Level of Cultured Osteoblasts in Rats. Sheng Li Xue Bao 57, 384–388. PubMed Abstract | Google Scholar 15968437

[B50] Watanabe Y. Ohshima H. Mizuno K. Sekiguchi C. Fukunaga M. Kohri K. (2004). Intravenous Pamidronate Prevents Femoral Bone Loss and Renal Stone Formation during 90-day Bed Rest. J. Bone Miner Res. 19, 1771–1778. 10.1359/jbmr.040811 PubMed Abstract | 10.1359/jbmr.040811 | Google Scholar 15476576

[B51] Winkelman C. (2009). Bed Rest in Health and Critical Illness. AACN Adv. Crit. Care 20, 254–266. 10.1097/nci.0b013e3181ac838d PubMed Abstract | 10.1097/nci.0b013e3181ac838d | Google Scholar 19638747

[B52] Winslow E. H. (1985). Cardiovascular Consequences of Bed Rest. Heart Lung 14, 236–246. 10.1097/00000637-198501000-00017 PubMed Abstract | 10.1097/00000637-198501000-00017 | Google Scholar 3852815

[B53] Worcester E. M. Beshensky A. M. (1995). Osteopontin Inhibits Nucleation of Calcium Oxalate Crystals. Ann. NY Acad. Sci. 760, 375–377. 10.1111/j.1749-6632.1995.tb44661.x PubMed Abstract | 10.1111/j.1749-6632.1995.tb44661.x | Google Scholar 7785921

[B54] Zuccarelli L. Baldassarre G. Magnesa B. Degano C. Comelli M. Gasparini M. (2021). Peripheral Impairments of Oxidative Metabolism after a 10‐day Bed Rest Are Upstream of Mitochondrial Respiration. J. Physiol. 599, 4813–4829. 10.1113/jp281800 PubMed Abstract | 10.1113/jp281800 | Google Scholar 34505290 PMC9293208

